# Natural History of Patients With Mitochondrial ATPase Deficiency Due to Pathogenic Variants of MT-ATP6 and MT-ATP8

**DOI:** 10.1212/WNL.0000000000213462

**Published:** 2025-03-20

**Authors:** Sara Carli, Anna Levarlet, Daria Diodato, Enrico Silvio Bertini, Diego Martinelli, Alessandro Malandrini, Diego Lopergolo, Gian Nicola Gallus, Rebecca D. Ganetzky, Chiara La Morgia, Valerio Carelli, Guido Primiano, Cristina Domínguez-González, Pablo Serrano-Lorenzo, Miguel A. Martín, Anna Ardissone, Costanza Lamperti, Valeria Nicoletta, Thomas Klopstock, Felix Distelmaier, Leopold Zeng, Boriana Büchner, Michelangelo Mancuso, Markus Schuelke, Alessandro Prigione, Caterina Garone

**Affiliations:** 1 Department of Medical and Surgical Sciences, Alma Mater Studiorum, University of Bologna, Italy;; 2 Unit of Neuromuscular and Neurodegenerative Disorders, Bambino Gesù Children's Research Hospital IRCCS, Rome, Italy;; 3 Department of Medicine, Surgery and Neurosciences, University of Siena, Italy;; 4 Division of Human Genetics, Children's Hospital of Philadelphia, Department of Pediatrics, University of Pennsylvania Perelman School of Medicine, PA;; 5 Department of Biomedical and Neuromotor Sciences, University of Bologna, Italy;; 6 IRCCS Istituto delle Scienze Neurologiche di Bologna, Programma di Neurogenetica, Bologna, Italy;; 7 IRCCS Istituto delle Scienze Neurologiche, UOC Clinica Neurologica, Bologna, Italy;; 8 Dipartimento di Neuroscienze, Organi di Senso e Torace, Fondazione Policlinico Universitario Agostino Gemelli IRCCS, Rome, Italy;; 9 Neurology Department. Neuromuscular Unit, ERN-NMD. Hospital Universitario 12 de Octubre, Madrid, Spain;; 10 Mitochondrial and Neuromuscular Disorders Group, Hospital Universitario 12 de Octubre, imas12 Research Institute, Madrid, Spain;; 11 Centro de Investigación Biomédica en Red de Enfermedades Raras (CIBERER), Madrid, Spain;; 12 Neuropschiatric Unit, Fondazione IRCCS Istituto Neurologico Carlo Besta, Milan, Italy;; 13 Department of Neurology, Friedrich-Baur-Institute, University Hospital, Ludwig-Maximilians-Universität (LMU) München, Munich, Germany;; 14 German Center for Neurodegenerative Diseases (DZNE), Munich, Germany;; 15 Munich Cluster for Systems Neurology (SyNergy), Germany;; 16 German Network for Mitochondrial Disorders (mitoNET), Munich, Germany;; 17 Department of General Pediatrics, Neonatology and Pediatric Cardiology, Medical Faculty, Heinrich-Heine-University Düsseldorf, Germany;; 18 Department of Clinical and Experimental Medicine, Neurological Institute, University of Pisa and AOUP, Italy;; 19 Department of Neuropediatrics, Charité-Universitätsmedizin Berlin, Corporate Member of Freie Universität Berlin, Humboldt-Universität Berlin, and Berlin Institute of Health, Germany;; 20 Department of General Pediatrics, Neonatology and Pediatric Cardiology, Medical Faculty, University Hospital Düsseldorf, Heinrich Heine University Düsseldorf, Germany; and; 21 UOC Neuropsichiatria dell'Età Pediatrica, IRCCS Istituto delle Scienze Neurologiche di Bologna, Bologna, Italy.

## Abstract

**Background and Objectives:**

The mitochondrial DNA (mtDNA) genes *MT-ATP6* and *MT-ATP8* encode for subunits α and 8 (A6L) of the adenosine triphosphate synthase complex. Pathogenetic variants in *MT-ATP6/8* cause incurable mitochondrial syndromes encompassing a wide spectrum of clinical features including ataxia, motor and language developmental delay, deafness, retinitis pigmentosa, and Leigh pattern in brain MRI. Typically, higher levels of mtDNA variants lead to more severe symptomatology although even individuals with similar mtDNA mutational loads exhibit high clinical variability. Hence, the establishment of potential therapeutics is currently challenging. In this article, we present an international multicenter study designed to provide a retrospective natural history of patients with MT-ATP6/8 deficiency and to identify primary and secondary end points for future clinical trials.

**Methods:**

Clinical, biochemical, and molecular genetics data of patients with genetically confirmed *MT-ATP6/8* defects were collected and analyzed from Italian, German, US, and Spain national reference centers through ethical committee–approved mitochondrial patients' national registries or local programs.

**Results:**

A cohort of 111 patients, 98 unreported, were analyzed (55 male, 56 female). Patients had infantile-onset disease (<1 year) in 44% of cases, pediatric-onset (≥1 year and ≤12 years) in 36%, and late-onset (>12 years) in 20%. Kaplan-Meier analysis showed a significant difference (*p* value = 0.0349) in the survival of infantile and pediatric patients compared with adult patients, although only 8% of patients were not alive at the last follow-up. The CNS was the most frequently affected tissue (93%), followed by the muscle (75%), eye (46%), and heart (18%). Brain MRI showed isolated Leigh-like lesions (58%), Leigh-like lesions and cortical and/or cerebellar atrophy (15%), isolated cerebellar atrophy (10%), and other lesions (21%). At the last follow-up, 11% of patients were wheelchair-bound. Metabolic acidosis or acute deterioration complicated the clinical course in ≅55% of early-onset patients. Molecular genetics studies identified 26 pathogenic variants (6 of them novel). Reduced citrulline levels and increased alanine and lactate levels were reported in 56%, 49%, and 71% of patients, respectively, suggesting their role as potential biomarkers.

**Discussion:**

Our results define a more accurate classification based on the age at onset for MT-ATPase deficiency and provide fundamental clinical and biochemical data for disease management.

## Introduction

Mitochondria play a fundamental role in central cellular functions by sustaining aerobic respiration through the production of cellular adenosine triphosphate (ATP). The process is permitted through oxidative phosphorylation (OXPHOS), which is performed by the electron transport chain complexes I to IV and the ATP synthase.^1^ ATP synthase, also known as complex V (CV), is a bioenergetic pump located within the mitochondrial inner membrane, and it catalyzes the last step of the OXPHOS permitting the conversion of adenosine diphosphate and inorganic phosphate (Pi) into ATP. CV is composed of 18 protein subunits, 16 encoded by the nuclear DNA and two by the mitochondrial DNA (mtDNA) (*MT-ATP6* and *MT-ATP8*),^2-4^ organized in 2 domains: F_1_, the catalytic domain bound to the inner mitochondrial membrane, and F_o_, the membrane domain situated in the mitochondrial matrix.

MT-ATP6 is a hydrophobic polypeptide composed of 226 amino acids while MT-ATP8 is a small hydrophilic polypeptide encompassing 68 amino acids that associates the membrane portion of the enzyme with the F_1_ catalytic domain. The 2 coding genes share a 46-nucleotide overlapping region between positions 8527 and 8572.^5^ Pathogenic variants in both genes share the same disease mechanism and functional consequences, and they have been previously associated with a wide spectrum of disorders, including neuropathy, ataxia, retinitis pigmentosa (NARP), cardiomyopathies, maternally inherited diabetes and deafness, maternally inherited Leigh syndrome (MILS), lactic acidosis, hypotonia, and developmental delays.^6^ Clinical heterogeneity is further complicated by heteroplasmy defined by the coexistence of wild-type and mutated mtDNA molecules within the same cells. Consequently, the clinical manifestations and their severity vary according to the mtDNA mutational load in the different tissues,^7^ thus challenging an accurate prognosis of the disease. *MT-ATP6* gene defect was the more common molecular diagnosis in reported mtDNA-related pediatric large cohort series associated with different clinical pictures.^8^

Natural history studies have been fundamental in revising clinical classification; analyzing morbidity and mortality; and identifying clinical, molecular, and biochemical parameters for clinical trial design in other mitochondrial disorders.^9,10^ Several cohorts of patients with *MT-ATP6* defect have been previously characterized, but no data are available for the natural history of this disorder.^11-13^

In this article, we present an international multicenter study aiming to provide a retrospective natural history of MT-ATPase deficiency in a cohort of 111 genetically confirmed patients with a diagnosis of MT-ATP6/8 deficiency.

## Methods

The multicentric study was conducted by selecting patients with confirmed genetic diagnosis of MT-ATP6/8 deficiency from the German (mitoNet) and Italian (MITOCON and MIRE2020) registries for patients with confirmed mitochondrial disorders and through the collaboration of physicians from mitochondrial disease research centers in Spain (Hospital Universitario 12 de Octubre) and the United States (Children's Hospital of Philadelphia) who enrolled patients in dedicated programs for mitochondrial disorders as specified in the Standard Protocol Approvals section. In addition, clinicians caring for already reported patients were contacted to provide follow-up information for published patients or to contribute to the study with additional unreported patients with a confirmed molecular diagnosis of MT-ATP6/8 deficiency.

The following data of patients were extrapolated from the databases and analyzed: demographics (sex and ethnicity), age at onset, age at the last follow-up, survival, cause of death, symptoms/signs of cardiac involvement (cardiomyopathy, arrhythmia, heart failure, heart attack, acute arrhythmia), central and peripheral nervous system involvement (movement disorders, dysmetria, ataxia, dystonia, hypotonia, cognitive impairment, motor impairment, nystagmus, lethargy, and peripheral neuropathy), muscular signs and symptoms (weakness/fatigue, ptosis, myalgia, dysphonia, and dysphagia), ocular sign and symptoms (visual defect, retinitis pigmentosa, optic atrophy, ophthalmoplegia), onset systemic features (metabolic acidosis, feeding difficulties, hypothermia, and others), complications during the clinical course (episodes of acute deterioration), need for medical procedures (ventilatory support and nutrition through nasogastric tubes or gastrostomy), the loss of the ability to walk, and instrumental investigations (brain MRI). Laboratory variables collected include serum creatine kinase (CK), plasma amino acids (citrulline and alanine), mitochondrial marker growth differentiation factor-15, and plasma lactic acid. The definition of N/A was used when data are not accessible or cannot be obtained for a particular query. The term “infantile” was used when age at onset was <1 year; “pediatric” when ≥1 year and ≤12 years; and “late onset” when >12 years. Direct Sanger sequencing or next-generation sequencing of whole-exome, whole-mitochondrial genome, or targeted gene panel libraries was used for molecular genetic studies of the *MT-ATP6* and *MT-ATP8* genes (NC_012920.1). Data on heteroplasmy levels of mtDNA were collected from blood, muscle, and urine.

### Standard Protocol Approvals, Registrations, and Patient Consents

Informed consent for the anonymous publication of the patient's clinical, biochemical, histologic, and molecular genetics data was obtained from all study participants under the local ethics committee approval of the referring clinical center at the time of the diagnosis workup and/or when enrolled in the national registries or local studies. In detail, patients were recruited in Italian and German reference centers for mitochondrial disorders in the national Italian (Mitocon and Mire2020) and German (MitoNET) registries for patients with confirmed diagnoses of mitochondrial diseases. All the reference centers received ethical committee approval for the mitochondrial patients' registry. Retrospective data were collected from dedicated databases that are harmonized under the GENOMIT project. Anonymized data from deceased patients were also included. Spanish patients were recruited by the national reference center for mitochondrial disorders at the Hospital Universitario 12 de Octubre in a retrospective study approved by the local ethics committee; US patients were enrolled at Children's Hospital of Philadelphia Institute in a dedicated program for mitochondrial patients approved by the institutional ethical committee (IRB 08–006177) and data collected in a Redcap database.

### Statistical Analysis

All statistical analyses were performed using GraphPad Prism 10.2. Results were expressed as a ratio and percentage of available data. Kaplan-Meier survival analysis was performed using the log-rank test (Mantel-Cox test).

## Data Availability

The anonymized data can be accessed on reasonable request addressed to the corresponding author.

## Results

### Clinical Features

Data were gathered from a cohort of 111 patients genetically diagnosed with MT-ATP6 and MT-ATP8 deficiency (56 male and 55 female). Among them, 98 patients were not previously reported in the literature and 13 were updated patients. The age at onset was available for 99 patients (12 N/A), and it ranged from birth to 58 years (average age: 7.8 years, median age: 1 year).

**Figure 1:**
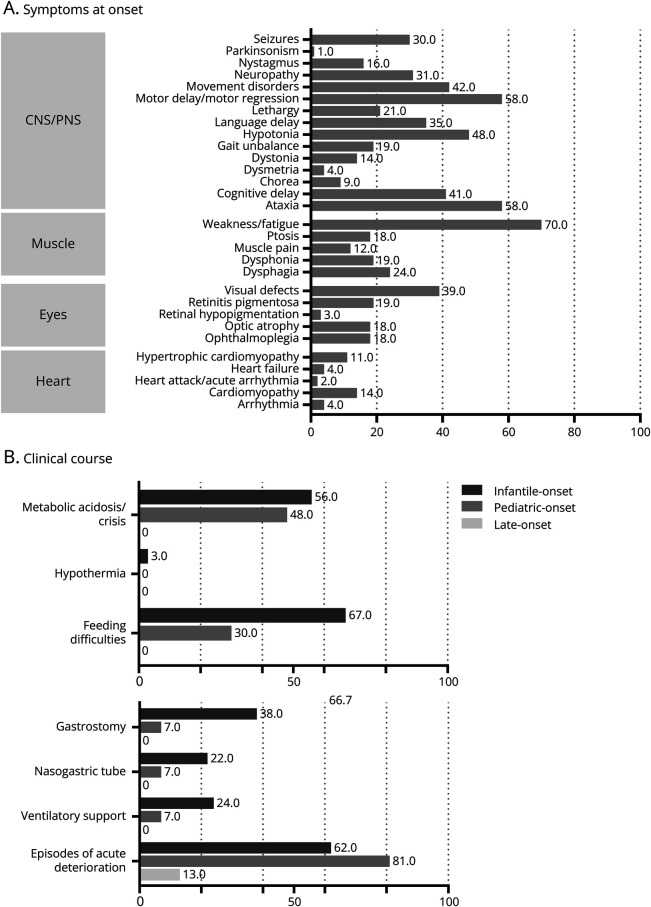
MT-ATPase Deficiency Clinical Phenotype (A) Clinical signs and symptoms at the disease onset (data available for 97 patients in heart, 111 in CNS, 104 in muscle, 99 in eyes); (B) Critical care needs and acute events during the clinical course. Data are represented as bar graphs and expressed in percentages (data available for 36 infantile-onset patients, 28 pediatric-onset patients, and 15 late-onset patients).

The central and peripheral nervous system (CNS/peripheral nervous system [PNS]), muscles, eyes, and heart were the principally affected organs. Cardinal signs and symptoms are graphically presented in Figure 1A.

CNS and PNS were affected in 103 of 111 patients (93%). Nystagmus was present in 18 patients (16%); twelve of them showed horizontal nystagmus, while in single patients, it was rotational, end-gate, and gaze-evoked nystagmus.

Brain MRI was performed in 86 patients, and 78 of 86 (91%) showed the following abnormalities: Leigh-like lesions were present in 42 of 78 (54%), Leigh-like lesions with additional cortical and/or cerebellar atrophy in 12 of 78 (15%), isolated cerebellar atrophy in 8 of 78 (10%), and other lesions (white matter abnormalities, cortical atrophy, cortical dysplasia, hydrocephalus) in 16 of 78 (21%). Four patients (6%) underwent brain magnetic resonance spectroscopy, and a lactate peak was found.

Muscle involvement defined by the presence of muscular signs/symptoms was reported in 78 of 104 patients (75%). No data were available for 7 patients. Among 70 patients with available data, 45 (64%) maintained the ability to walk while global motor functions were lost in 12 patients (17%) and 8 patients (11%) were wheelchair-bound at the last follow-up. In addition, 13 patients never acquired the ability to walk (19%), 10 with infantile onset and 3 with pediatric onset. Among early-onset patients, 1 infantile-onset and 3 pediatric-onset patients experienced periodic paralysis after an acute metabolic crisis or infection. Periodic paralysis was observed also in a patient with no available age at onset. Muscle histology was analyzed in 13 patients, and it did not reveal abnormal patterns, except for 2 patients with mild neurogenic pathologic changes.

The ocular system was affected in 46 of 100 patients (46%, 11 N/A) while one or more signs/symptoms of cardiac involvement were reported in 17 of 97 patients (18%) (14 N/A).

Additional signs/symptoms were metabolic acidosis (35/83, 42%) and feeding difficulties (33/83, 40%), present only in the early-onset patients (28 N/A). Hypothermia was reported in an isolated infantile-onset patient (1/83, 1%). Follow-up data from the clinical course were available in 83 patients, and they included episodes of acute deterioration (49/83, 59%), ventilatory support (11/83, 13%), and nutritional support (27/83, 33%). The acute deterioration was reported after one or more episodes of viral illness in 12 patients (24%), high fever in 6 (12%), metabolic crisis in 6 (12%), gastroenteritis in 2 (4%), and pneumonia in 2 (4%), while in 2 patients, there was no report of a specific trigger (4%). Among patients who experienced acute deterioration, 5 patients had died.

Laboratory tests revealed a decrease in citrulline levels in 20 of 36 patients with available data (56%) (average value 5.3, normal reference value: 20–34 mmol/L). Thirteen patients belonged to the infantile-onset subgroups and 6 to the pediatric subgroup, while for a single patient, no age at onset was available. Alanine levels were increased in 17 of 35 patients (49%) with an average level of 655.3 mmol/L compared with the normal reference range of 253–427 mmol/L. Most of the patients showing altered levels belonged to the infantile-onset group (12/17) or the pediatric one (4/17), whereas just a single patient of the late-onset group presented an alanine increase. The age at onset was not available for 3 patients. CK was increased in 15 of 54 patients with available data (28%). Six had infantile onset, 4 pediatric onset, and 2 late onset (3 N/A). Plasma lactic acidosis (normal reference value: 5–22 mg/dL) was detected in 49 of 69 patients (71%) (42 N/A), stratified in 28 patients with infantile onset, 16 with pediatric onset, 3 with late onset, and 2 with unavailable age at onset. The growth/differentiation factor-15 (GDF-15) level was available in a small subgroup of patients and was deregulated in 4 of 21 patients (19%) (average level 990 pg/mL).

### Clinical Classification

Based on the previously described clinical syndromes associated with *MT-ATP6/8* defects, 3 patients (3%) met the criteria for CPEO + syndrome (chronic progressive external ophthalmoplegia with multisystemic features), 10 patients (9.0%) for NARP syndrome (NARP), 9 patients (8%) for classic Leigh syndrome (LS) with exclusively neurologic signs/symptoms, and 45 patients (41%) for Leigh-like syndrome with multisystemic involvement (LS+) while 44 patients (40%) presented with miscellaneous other phenotypes.

Patients with NARP syndrome (10/111) had infantile onset in 22% of cases, pediatric onset in 22%, and late onset in 56%. They showed additional signs such as visual defects (9/10, 90%), optic atrophy (4/10, 40%), weakness (7/10, 70%), muscle pain (2/10, 20%), cognitive (3/10, 10%) and motor (2/10, 20%) impairment, and hypotonia (2/10, 20%). In addition, there were isolated patients with cardiomyopathy, arrhythmia, dystonia, nystagmus, seizures, and dysphagia.

Patients with LS had infantile (86%) or pediatric (14%) onset. They reported cognitive impairment (100%), motor delay (7/9, 78%), seizures (5/9, 56%), hypotonia (4/9, 44%), movement disorders (3/9, 33%), ataxia (2/9, 22%), and lethargy (2/9, 22%).

Patients with Leigh-like syndromes with multisystemic involvement (45/111) mostly had infantile (45%) or pediatric (43%) onset while only 11% had a late onset. They manifested additional neuromuscular signs/symptoms (41/44, 93%, 1 N/A) such as weakness (37/44, 84%), dysphagia (16/44, 36%), dysphonia (12/44, 27%), and ptosis (14/44, 32%); ocular involvement (18/39, 46%, 6 N/A) and specifically visual defects (15/39, 38%), ophthalmoplegia (11/39, 28%), optic atrophy (7/39, 18%), retinitis pigmentosa (5/39, 13%), and retinal hypopigmentation (2/39, 5%); and cardiac signs/symptoms (11/40, 27%, 5 N/A).

Considering the clinical variability of our cohort and the number of patients who do not fulfill the criteria for a diagnosis of NARP or LS, we stratified our cohort based on their age at onset. The 3 subgroups were infantile onset (<1 year, 43/99, 44%), pediatric onset (≥1 year and ≤12 years, 36/99, 36%), and late onset (>12 years, 20/99, 20%). Symptoms and signs at the disease onset and during the clinical course are presented in Figure 1B and Table 1.

**Table 1:** Percentage of Signs and Symptoms According to the Age at Onset

	Age at onset (%)		
Sign/symptom	Infantile	Pediatric	Late
Central/peripheral nervous system			
Ataxia	28	89	85
Cognitive delay/language delay	74	72	20
Dysmetria	2	3	10
Dystonia	14	19	5
Hypotonia	67	50	15
Lethargy	30	14	15
Motor delay/motor regression	81	61	20
Movement disorders	30	64	50
Neuropathy	14	36	70
Nystagmus	16	14	20
Seizures	47	22	5
Muscle			
Dysphagia	32	26	16
Dysphonia	10	34	21
Muscle pain	0	9	47
Ptosis	20	20	21
Weakness/fatigue	59	83	95
Eyes			
Ophthalmoplegia	21	12	32
Optic atrophy	10	18	37
Retinal hypopigmentation	0	6	5
Retinitis pigmentosa	10	15	42
Visual defects	38	24	63
Heart			
Arrhythmia	3	3	11
Cardiomyopathy	29	9	5
Heart attack/acute arrhythmia	0	3	5
Heart failure	8	3	0
Hypertrophic cardiomyopathy	21	0	0

Patients were stratified as follows: infantile onset (<1 y, 43/99, 44%), pediatric onset (≥1 y and ≤12 y, 36/99, 36%), and late onset (>12 y, 20/99, 20%). Data are presented as a percentage of the available data.

### Critical Care Needs

During the clinical course, 23 of 37 infantile patients (6 N/A) manifested episodes of acute deterioration (62%). Ventilatory support was required in 9 of 37 patients (24%) and nutritional support with gastronomy or nasogastric tubes in 14 (38%) and 8 (22%), respectively.

For the pediatric subgroup, episodes of acute deterioration were reported in 22 of 27 patients (81%) (9 N/A). Ventilatory support was required in 2 of 27 patients (7%) while nutritional support with gastrostomy or nasogastric tubes in 7% and 7%, respectively.

Only 2 of 15 of the late-onset patients manifested episodes of acute deterioration (13%) (5 N/A). Ventilatory and nutritional support were not required for this group.

### Survival Rate

**Figure 2:**
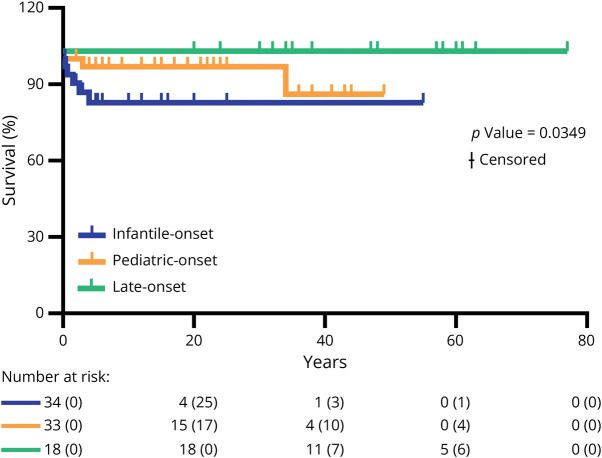
Survival Curves Kaplan-Meier analysis of the survival in the 3 age groups (n = 91 individuals: 34 with infantile onset, 33 with pediatric onset, and 18 with late onset). Survival analysis was assessed by the log-rank Mantel-Cox test. Numbers at risk are specified below each panel.

Follow-up data were available for 91 patients: 34 infantile-onset, 33 pediatric-onset, and 18 late-onset (6 N/A). The survival rate was not severely reduced in our cohort, with 84 patients still alive (92%) at the last follow-up. In the infantile-onset group, follow-up spanned from 4 months to 55 years (average 7.8 years; median 4 years); in the pediatric-onset group, spanned from 2 years to 49 years (average: 20.4 years; median: 19 years); and in the late-onset group, spanned from 20 years to 77 years (average: 46.7 years; median: 48 years). Deceased patients belonged to the infantile-onset (5) and the pediatric-onset (2) groups while the 18 patients of the late-onset group were all alive at the last follow-up. When we compared the overall survival rate in the 3 age-at-onset groups, we found statistical differences (*p* value = 0.0349 by Mantel-Cox test, Figure 2) with the highest mortality in the infantile-onset group. In particular, the infantile-onset group showed an almost significant difference in the survival rate when compared with pediatric-onset or late-onset groups (*p* value = 0.0811 and 0.0680, respectively) while only a tendency was observed by comparing pediatric-onset and late-onset groups (*p* value = 0.1570). The causes of death were pneumonia (1/7, 14%), respiratory insufficiency (1/7, 14%), seizures (2/7, 29%), COVID-19 complications (1/7, 14%), multiorgan failure during a metabolic crisis (1/7, 14%), and severe cardiomyopathy (1/7, 14%). There was no difference in survival when we compared NARP, LS, Leigh-like syndrome, or miscellaneous groups (*p* value = 0.0559, eFigure), although 5 of 7 deceased patients belonged to the subgroup of Leigh-like syndrome with multisystemic involvement, one to the classic LS subgroup, and one to the miscellaneous subgroup. The post hoc test in survival rates highlighted a statistical difference in patients with LS vs miscellaneous group (*p* value = 0.0196) and a tendency in the survival curve comparison among patients with multisystemic LS and the miscellaneous group (*p* value = 0.0621).

### Molecular Genetics

**Figure 3:**
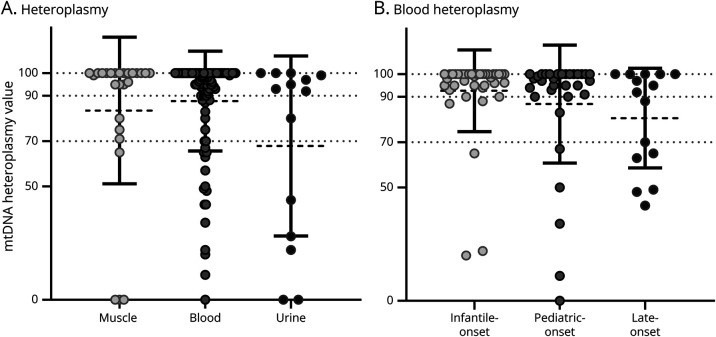
MT-ATP6/8 Heteroplasmy Levels (A) Scattered plots showing heteroplasmy levels in blood (n = 95), muscle (n = 26), and urine (n = 13), in the overall cohort. (B) Heteroplasmy levels in blood in the 3 age groups (n = 38 with infantile onset; 32 with pediatric onset; 15 with late onset).

The degree of heteroplasmy levels was determined in muscle (26 patients), blood (96 patients), and urine (14 patients) (Figure 3A and Table 2). It is important to note that in the blood, the degree of heteroplasmy grossly correlates with the age at onset with a mean value of 92.5% in infantile-onset patients, 86.8% in pediatric-onset, and 80.6% in late-onset (Figure 3B), although it did not reach the statistical significance. No correlation with the age at onset was found for mtDNA levels measured in muscle tissue or urine.

**Table 2:** Heteroplasmy Levels in Different Organs/Tissues

							Number of patients (mean value)		
Tissue	N	Range	Median	Mean	Degree (%)		Infantile	Pediatric	Late
Muscle	26	0–100	100	83.5	≤70	15	9 (95.7)	9 (81.7)	4 (75)
					>70 and < 90	12			
					≥90	19			
					Homoplasmic	54			
Blood	95	0–100	97	87.5	≤70	18	39 (92.5)	32 (86.8)	15 (80.6)
					>70 and < 90	10			
					≥90	29			
					Homoplasmic	43			
Urine	14	0–100	92.5	67.9	≤70	36	4 (88.0)	4 (64.0)	3 (97.3)
					>70 and < 90	7			
					≥90	36			
					Homoplasmic	21			

Data on age at onset were not available for 4 patients in muscle, 10 patients in blood, and 3 patients in urine.

**Figure 4:**
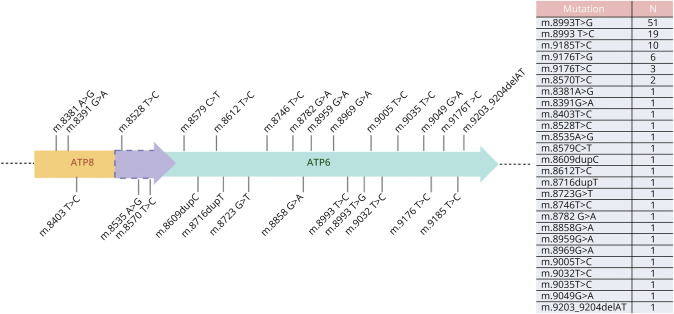
Graphical Representation of *MT-ATP6* and *MT-ATP8* Genes With the Identified Variants in Our Cohort

Genetic analyses identified 26 pathogenic variants, 6 of them not previously reported in the literature (Figure 4). Among unreported variants (eTable), 2 were carried by patients with infantile onset (m.8579 C > T, m.8716dupT) and 3 with pediatric onset (m.8391 G > A, m.8535 A > G, m.8858 G > A) while for patients carrying the m.9203_9204delAT variant, the age at onset was not available.

Among variants, 3 were in the *MT-ATP8* gene, 3 in the overlapping region, and 20 in the *MT-ATP6* gene. The most frequent variants of the *MT-ATP6* gene were as follows: (1) the m.8993T > G, found in 51 patients (46%), 28 of them with infantile onset (61%), 10 with pediatric onset (22%), and 8 with late onset (17%) (age at onset not reported for 5 patients); (2) m.8993T > C, present in 19 patients (17%); (3) m.9185T > C, present in 10 patients (9%). Both m.8993T > C and m.9185T > C were equally distributed among the 3 classification groups.

## Discussion

Pathogenetic variants in *MT-ATP6* were first described in patients with NARP syndrome/MILS.^14,15^ Since then, over 300 patients have been reported in literature,^12,16,17^ demonstrating a broader and more variable phenotype. *MT-ATP8* variants or variants in the overlapping regions between *MT-ATP6* and *MT-ATP*8 are instead more recent discoveries with a few reported patients in literature with a phenotype including cardiomyopathy, diabetes, encephalopathy, peripheral neuropathy, and progressive external ophthalmoplegia.^18-21^ The overall clinical variability of MT-ATP6/8 deficiency challenges the design of clinical trials and the development of novel experimental therapy.

In this study, we retrospectively collected data from a cohort of 111 patients with MT-ATP6/8 deficiency, including 98 previously unreported patients, with the main goal of analyzing morbidity, mortality, and other clinical and biochemical parameters useful for clinical trial design.

In our cohort, most patients presented Leigh-like syndrome with multisystemic involvement (41%) or other miscellaneous phenotypes (40%) and only a few patients fulfilled the criteria for NARP (9%), classic LS (8%), or CPEO + syndrome (3%). Our data confirm *MT-ATP6/8* defect as prevalently associated with LS, as reported in previously described large cohort studies.^22^ The prevalence of NARP syndrome in our cohort was comparable with the results of a previous clinical-genetics study^12^ in a cohort of patients with *MT-ATP6* variants that showed only 8% of patients with NARP. However, our study did not include unaffected relatives and showed a higher clinical heterogeneity. To better understand the natural history of the disease, we classified our patients according to the age at onset, by identifying 3 different subgroups: infantile onset (age at onset <1 year, 44%), pediatric onset (≥1 year and <12, 36%), and late onset (≥12 years, 20%). Our data demonstrated that *MT-ATP6/8* defects present as a metabolic encephalomyopathy in early-onset patients, with 56% of infantile patients with MT-ATPase deficiency and 48% of pediatric patients with MT-ATPase deficiency experiencing episodes of metabolic acidosis. The clinical course was complicated by acute deterioration in 62% of infantile-onset patients and 81% of pediatric-onset patients. Critical care needs such as ventilatory and nutritional support were required, respectively, in 24% and 60% of infantile patients whereas less frequently in pediatric patients (7% and 15%). Those characteristics were distinctive for early-onset patients. By contrast, late-onset patients with MT-ATPase deficiency never experienced metabolic acidosis nor required ventilatory and nutritional support and only 2 patients reported acute deterioration events.

Laboratory test results are in line with this observation by demonstrating reduced levels of citrulline in 56% of patients and an increase in alanine levels in 49% of infantile-onset and pediatric-onset patients, suggesting them as biomarkers for early-onset patients. Of interest, reduced levels of plasma citrulline were found at newborn screening in 6 patients subsequently genetically diagnosed with *MT-ATP6* variants and treated with citrulline supplementation that prevented metabolic crisis and reduced the severity of the disease.^17^ Therefore, citrulline might be considered both a biomarker and supportive treatment for patients with MT-ATPase deficiency. Further prospective studies are needed to confirm this hypothesis.

Plasma lactic acid and creatine phosphokinase were instead increased in all age groups in our cohort.

Our study demonstrated high morbidity of mitochondrial *MT-ATP6/8* defect but low mortality. Contrarily to other mitochondrial disorders presenting a rapid progression to death, 92% of patients were still alive at the last follow-up and survival was reduced only in a subgroup of infantile (5) and pediatric (2) patients. Our results also showed higher survival compared with a previous MT-ATP6 cohort study^12^ or natural history studies on LS including patients with *MT-ATP6* defects, reporting a survival between 64% and 74% in patients.^11^ However, those data have the limit of being retrospectively collected, and prospective studies are needed to confirm the survival rate of patients with MT-ATP6/8 defect.

MT-ATPase deficiency is in any case a severely debilitating disease affecting motor and visual functionality.

Muscle weakness or fatigue was reported in 70%, causing a moderate to severe degree of reduced motor functionality: 19% never acquired the ability to walk, global motor functions were lost during the follow-up in 17%, and 11% of patients became wheelchair-bound.

Although the central and peripheral nervous system, heart, muscle, and eyes were affected in all age groups, we identified some distinctive features: cognitive impairment was one of the cardinal signs in both infantile-onset and pediatric-onset patients while absent in late-onset patients; cardiomyopathy was more prevalent in the infantile-onset group and progressively reduced in pediatric and adult patients while neuropathy/polyneuropathy and ataxia were predominant in late-onset patients.

The m.8993T > G variant was confirmed the most frequent in our cohort, being found in 46% and more prevalently but not exclusively associated with infantile MT-ATPase deficiency (61%). In addition, 22% of pediatric-onset patients and 17% of late-onset patients had the same variant. The m.8993T > C variant was present in 17% of patients, and the m.9185T > C was present in 9%, but both were equally distributed among the 3 classification groups. Therefore, we did not find a clear genotype-phenotype correlation as previously suggested by Stendel et al.^12^ with the specific variants. Although not statistically significant, we observed instead a correlation between heteroplasmy level in blood and the age at onset.

A recent repurposing drug screening study on neural progenitor cells derived from induced pluripotent stem cells suggested the benefit of the phosphodiesterase type 5 inhibition (PDE5i) by reducing mitochondrial membrane hyperpolarization and partially rescuing the altered mitochondrial Ca^2+^ homeostasis.^23^ Thanks to our efforts, the Committee for Orphan Medicinal Products of the European Medicines Agency assigned the orphan drug designation (ODD) to the PDE5i sildenafil for the treatment of LS (EU/3/23/2831). Another ODD was assigned to cannabidiol for LS (EU/3/23/2800). However, clinical trials will be fundamental for demonstrating the efficacy and safety of these possible new treatments.^24^

In summary, our study demonstrates the importance of classifying MT-ATPase deficiency based on the age at onset. Moreover, we identified plasma alanine and citrulline as metabolic biomarkers for early-onset patients and demonstrated the recurrence of acute deterioration and metabolic acidosis in pediatric and infantile patients. Finally, we identified the level of mtDNA heteroplasmy in blood as a possible prognostic factor. These data, together with the survival and morbidity of the disorders, represent fundamental information for designing a successful clinical trial.

## Glossary

ATP: adenosine triphosphate
CK: creatine kinase
COMP: Committee for Orphan Medicinal Product
CV: complex V
ISCIII: Instituto de Salud Carlos III
LS: Leigh syndrome
MILS: maternally inherited Leigh syndrome
mtDNA: mitochondrial DNA
NARP: neuropathy, ataxia, retinitis pigmentosa
ODD: orphan drug designation
OXPHOS: oxidative phosphorylation
